# Supplementary material for: Comprehensive Analysis of the Role of Fibrinogen and Thrombin in Clot Formation and Structure for Plasma and Purified Fibrinogen
Source: Biomolecules. 2024 Feb 16;14(2):230. doi: 10.3390/biom14020230 (PMC10886591; doi:10.3390/biom14020230)
Supplement: Supplementary file 1 [file biomolecules-14-00230-s001.zip › biomolecules-2833072-supplementary.pdf]

Supplemental Information

S.1 Extended Methods

S.1.1: SEM Imaging

Purified Fibrinogen:

To create the SEM samples, 50 µL fibrinogen solution containing the peak 1 fibrinogen and FXIIIa (both from Enzyme Research Labs, Indiana, IN, USA) was placed into the lid of a 600 µL microcentrifuge tube that contained 5 holes from an 18-gauge needle, sealed on the outside with parafilm. The solution was then topped with 50 µL solution containing human alpha thrombin (Enzyme Research Labs, Indianapolis, IN, USA), such that the final sample concentrations ranged from 1-10 mg/mL fibrinogen, 0.1-1 U/mL thrombin, with 25 Loewy-U/mL FXIIIa, as noted in the respective results, in HBS buffer (150 mM sodium chloride, 20 mM HEPES, pH 7.4), with 5 mM calcium chloride. The solution was pipetted up and down once to promote mixing and incubated for one hour at 37°C in a humidified container.

After polymerization, the parafilm was removed and the clot was washed in HBS buffer three times for twenty minutes each, then fixed overnight in 2% glutaraldehyde. After fixation, the clot was again washed in HBS buffer three times for twenty minutes each, then dried in a series of ethanol solutions (30, 50, 70, 90, 100, 100, and 100%) for 15 minutes each, followed by a solution of 50% ethanol/50% hexamethyldisilazane (HMDS) for 10 minutes, then 100% HMDS for 10 minutes. The HMDS was then mostly removed, leaving just what was left on top of the clot in the lid, and the clot was left overnight to dry.

The dried clot was removed from the microcentrifuge lid and mounted onto a stub, then sputter coated with palladium silver using a Denton Vacuum Desk V sputter coater for 40 seconds at 30 mA and imaged using a Zeiss EVO 10 scanning electron microscope with 15 kV at 21,940x magnification.

Plasma:

Respective concentrations of fibrinogen, thrombin (0.1-1U/mL) or tissue factor (75 pM), and calcium chloride (25 mM final concentration) SEM samples were created in etched Delrin molds with 50 µL each.

For increasing fibrinogen concentrations, plasma containing a starting concentration of either 2.7 or 2.9 mg/mL fibrinogen was diluted with fibrinogen-depleted plasma to achieve 1 mg/mL or spiked with commercially available high fibrinogen plasma (Aniara Diagnostica) to achieve 5 or

10 mg/mL. Fibrinogen-depleted plasma was created using 0.1% batroxobin and 40 mM $CaCl_2$ (final concentration). The 2.7 mg/mL plasma was human-pooled with IRB approved protocol; this plasma was used for the 1:80 increasing fibrinogen conditions for confocal and turbidity. The rest of the plasma experiments were performed with the 2.9 mg/mL plasma. The 2.9 mg/mL plasma was commercially purchased from Cone Bioproducts (catalog number 5781). For simplicity in the main text, we refer to the plasma concentration as 2.7 mg/mL. Human-pooled plasma was used to avoid variation from individual plasmas. For the first set of experiments, a fixed concentration of tissue factor (TF) (1:80 volume) was used. For the second set of experiments, all samples had a starting fibrinogen concentration of 2.7 or 2.9 mg/mL with 0.1, 0.25, 0.5, or 1 U/mL final thrombin concentrations. For the third set of experiments, the amount of TF was increased with increasing fibrinogen concentration to achieve a fixed ratio between the two. Clots were allowed to form for 30 minutes at at RT.

All samples were washed with sodium cacodylate for 1 hour, fixed with 2% glutaraldehyde overnight, and washed again with sodium cacodylate the following day. The clot was then placed in the same manner into solutions of 30%, 50%, 70%, 90%, 100%, 100%, and 100% ethanol, sequentially. Samples were then placed into a solution of 1:1 100% ethanol and hexamethyldisilazane for 10 minutes, then in 100% hexamethyldisilazane for 10 minutes. Samples were allowed to dry completely. The samples were mounted onto a stub with double-sided carbon tape and sputter coated with 15 nm of gold or gold/palladium using an EMS 150T ES. Samples were imaged using a Zeiss SIGMA field emission scanning electron microscope (FESEM) obtained with 5 kV at 20,000x magnification.

Analysis of SEM Samples:

There were at least two samples imaged per concentration. Fiber diameter values were manually obtained from the acquired SEM images using the line segment measuring tool in ImageJ (http://rsbweb.nih.gov/ij/). This process was repeated in the same manner for clots at each fibrinogen/thrombin concentration being analyzed.

S.1.2: Confocal Imaging

Purified Fibrinogen:

To prepare the confocal samples, 5 μL fibrinogen solution containing peak 1 fibrinogen, AlexaFluor-647-labeled-fibrinogen (Invitrogen, Eugene, OR, USA) (at a concentration of 1/65 that used of wild-type fibrinogen), and FXIIIa (in HBS buffer) was spread out onto a 22x22 mm cover glass. It was then topped with 5 μL of the appropriate concentration of thrombin solution, containing human alpha thrombin (Enzyme Research Labs, Indianapolis, IN, USA) in HBS buffer with 10 mM calcium chloride, and the solution was pipetted up and down once to promote mixing

of the thrombin and fibrinogen solutions. The sample was then placed into a humidified container to polymerize for one hour at 37°C. Following polymerization, the sample was topped with 5 μL HBS buffer and the cover glass was placed carefully onto a microscope slide, with the fibrin clot between the two, and sealed around the edges using fingernail polish. The same sample preparation was used for samples containing each of the fibrinogen/thrombin concentrations analyzed.

Confocal images were acquired on each sample using a Zeiss LSM800 laser scanning microscope, with a 63x (1.4 NA) oil immersion objective.

Plasma:

To prepare the confocal samples, fibrinogen, thrombin/TF, calcium, and AlexaFluor-488-labeled-fibrinogen (1% by volume) were mixed and placed in a 96-well black-walled plate. The samples were allowed to fully form at room temperature for 30 minutes. The same sample preparation was used for samples containing each of the fibrinogen/thrombin concentrations analyzed. Confocal images were acquired on each sample using a Zeiss LSM 800 confocal microscope (NA = 1.20), using a 40x water immersion or 63x oil immersion objective.

Analysis of Confocal Samples:

At least two samples were imaged for each investigated fibrinogen/thrombin concentration, and the acquired 2D images were used for analysis. The images were made binary using ImageJ, then both pore sizes and fiber lengths were measured manually using the line segment measuring tool in ImageJ (FIJI). To obtain the percentage of fluorescent area, considered to be the density, the area fraction tool in ImageJ was used.

S.1.3: Turbidity/Turbidimetry

Purified Fibrinogen:

A 96 well plate was prepared with three wells for each sample concentration being analyzed, three wells with background solution containing the same concentrations of buffer, thrombin, and FXIIIa as used in the sample solutions, but with no fibrinogen, and three wells with deionized water, all at a final volume of 150 μL per well. For the sample wells, 75 μL fibrinogen solution containing the peak 1 fibrinogen and FXIIIa in HBS buffer was loaded into the bottom of a 96 well plate. The fibrinogen solution was then topped with 75 μL thrombin solution containing human alpha thrombin (Enzyme Research Labs, Indianapolis, IN, USA) in HBS buffer with 10

mM calcium chloride. The final concentrations ranged from 1-10 mg/mL fibrinogen, 0.1-1 U/mL thrombin, with 25 Loewy-U/mL FXIIIa, as noted in the respective results.

Immediately after the addition of thrombin, the sample was shaken by the BioTek Synergy HT plate reader for 5 seconds, then “absorbance” measurements were taken every 15 seconds for one hour at a wavelength of 405 nm and a temperature of 37°C to capture the polymerization process. After that hour, absorbance measurements were taken on the same wells every 10 nm from 500-800 nm, as well as at a wavelength of 900 nm and 977 nm (in order to calculate the pathlength).

Plasma:

A 96 well plate was prepared with three replicates for each sample being analyzed, containing plasma, calcium, and thrombin or TF at their respective concentrations. A stock (400 μL) with all three materials were mixed together; 100 μL of plasma mix was added to each well. The plate was transferred to a SpectraMax plate reader. The “absorbance” measurements were taken every 15 seconds for one hour at a wavelength of 405 nm and a temperature of 37°C to capture the polymerization process. After that hour, absorbance measurements were taken on the same wells every 10 nm from 500-800 nm, as well as at a wavelength of 900 nm and 977 nm (in order to calculate the pathlength).

Analysis of Turbidity Curves:

To create the turbidity curves, the triplicate values for each sample were averaged, excluding any datasets that did not clot. The average absorbance value from the background wells were then subtracted from the average value for each sample of the corresponding thrombin concentrations for each time datapoint. The pathlength (in cm) was calculated by averaging the absorbance values for the wells with water and subtracting the value at 900 nm from the value at 977 nm, then dividing by 0.18 (the K-factor). To calculate the turbidity values (τ) at each timepoint for the samples, the absorbance values with the background subtracted out (A) were divided by the pathlength (x) and then multiplied by ln(10) such that:

$$\tau = \frac{A}{x} * ln\,(10).$$

Three parameters were analyzed from the turbidity assays: lag phase, maximum turbidity, and rate of clot formation. The lag phase is the time it takes for the clot to begin forming and was recorded as the closest value to a 0.1 increase in turbidity (cm^{-1}). Maximum turbidity was recorded for the normalized curves and assumed to represent a fully formed clot. Rate of clot formation was measured as the slope of the linear region from the lag phase to maximum turbidity.

There were multiple turbidimetry approaches used to determine fibrin diameter from light scattering measurements, as there are several different approaches commonly used within the fibrin community. We utilized the approaches introduced by Carr and Hermans [42], and the corrected approach introduced by Yeromonahos et. al. [43], as these approaches have been found to be reasonably accurate for purified fibrin under certain clot conditions [44].

For using the Carr-Hermans approach to determine fiber diameter from turbidimetry data, it was plotted as $\frac{\frac{dn}{dc}(\lambda)^2}{\langle\frac{dn}{dc}\rangle^2}\frac{n(\lambda)}{\langle n\rangle}\frac{c}{\tau\lambda^3}$ versus $\frac{n(\lambda)^2}{\langle n\rangle^2}\frac{1}{\lambda^2}$, where λ is the wavelength, $n(\lambda)$ is the refractive index of the solution, given by the equation $n(\lambda) = 1.3247 + \frac{3093.9}{\lambda^2}$ for purified fibrinogen [44], and $n(\lambda) = 1.3353 + \frac{4404.8}{\lambda^2} - \frac{9.1925*10^7}{\lambda^4}$ for plasma [82]. $\langle n\rangle$ is the spectrally averaged refractive index (1.33243 for purified fibrinogen and 1.34569 for plasma), $\frac{dn}{dc}(\lambda)$ is the specific refractive index increment of the solute in the solvent given by the equation $\frac{dn}{dc}(\lambda) = 0.1853 + \frac{1689.9}{\lambda^2}$ for purified fibrinogen, and $\frac{dn}{dc}(\lambda) = 0.18 + \frac{1169.9}{\lambda^2}$ for plasma (determined using the software SEDFIT [83,84]. $\langle\frac{dn}{dc}\rangle$ is the spectrally averaged specific refractive index increment (0.189525 for purified fibrinogen and 0.182925 for plasma), c is the fibrinogen concentration, and τ is the turbidity. The diameter was then determined by fitting the plot with a linear line of best fit and using the slope and y-intercept of the fit line to solve for the diameter such that:

$$d = \sqrt{\frac{77}{23}\frac{1}{\langle n\rangle^2\pi^2}\frac{slope}{yintercept}}$$

where the uncertainty in the diameter is given by:

$$\delta d = \sqrt{\left(\frac{1}{2}\left(\frac{77}{23}\frac{1}{\langle n\rangle^2\pi^2}\right)^{\frac{1}{2}}\left(\frac{slope}{yintercept}\right)^{-\frac{1}{2}}\frac{1}{yintercept}\right)^2 \delta slope^2 + \left(-\frac{1}{2}\left(\frac{77}{23}\frac{1}{\langle n\rangle^2\pi^2}\right)^{\frac{1}{2}} slope^{\frac{1}{2}} yintercept^{-\frac{3}{2}}\right)^2 \delta yintercept^2}$$

.

The Corrected Yeromonahos approach was also used to solve for the diameter by plotting the turbidimetry data as $\frac{\tau\lambda^5}{\frac{88}{15}\pi^3 n(\lambda)^3 \frac{c}{N_A}\frac{dn}{dc}(\lambda)^2}$ versus $\frac{\lambda^2}{n(\lambda)^2}$, where the variables are the same as defined above, and N_A is Avogadro's number. Fitting the plot with a linear line of best fit, the slope and y-intercept can be used to solve for the diameter such that:

$$d = 2\sqrt{\frac{-yintercept}{\frac{184}{231}\pi^2 slope}}$$

where the uncertainty in the diameter is:

$$\delta d = \sqrt{\left(-\left(\frac{1}{\frac{184}{231}\pi^2}\right)^{\frac{1}{2}}(-yintercept)^{\frac{1}{2}}(slope)^{-\frac{3}{2}}\right)^2 \delta slope^2 + \left(-\left(\frac{1}{\frac{184}{231}\pi^2}\right)^{\frac{1}{2}}(slope)^{-\frac{1}{2}}(-yintercept)^{-\frac{1}{2}}\right)^2 \delta yintercept^2}.$$

Statistical Analysis:

All statistical analysis was performed using GraphPad Prism 9.4.0. Outliers were identified and removed using the Grubbs' test with alpha of 0.05. To perform comparison tests on diameters measured using SEM images on the different conditions, normality was confirmed with the D'Agostino and Pearson test. Kruskal Wallis test was used for non-normal samples and Welch's corrected ANOVA was used for samples with different standard deviations. Formal comparison tests could not be performed on Carr-Hermans and Yeromonahos.

Unless otherwise specified, the following star nomenclature is used for statistical significance: ns not significant, * p<0.05, ** p<0.01, *** p<0.001, **** p<0.0001.

S.2. Turbidity and Turbidimetry Comparison Between ECU and RU Facilities

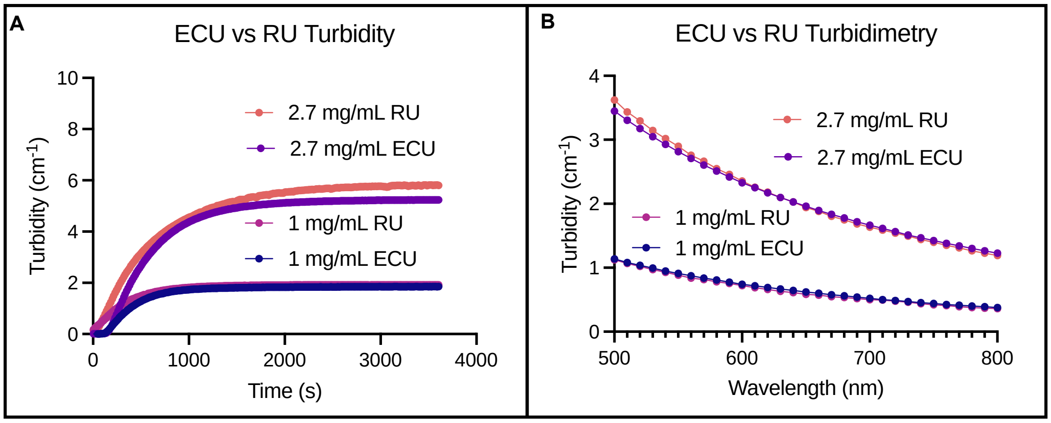

Fig. S.1. Turbidity and Turbidimetry Comparison Between ECU and RU Facilities. Comparison of turbidity and turbidimetry of clots made with purified fibrinogen when experiments were performed at ECU or RU using purified fibrinogen and the same FXIII and thrombin but university-specific protocols. This provides evidence that results are reproducible and are less dependent on technique specifics.

S.3 Room Temperature (RT) vs 37 Degrees Celsius

As noted in the methods, all polymerization steps were performed at 37℃ for purified fibrinogen; however, polymerization for plasma samples for confocal microscopy and SEM were performed at RT. To confirm this is an insignificant difference, we incubated clots at RT and 37℃ and

calculated diameter and % area. We observed no significance (p>0.05) in the diameter for either the purified fibrinogen or plasma clots, or in the fiber length for purified fibrinogen clots and % area for plasma clots. While there was a statistical difference in the pore size and % area for the purified fibrinogen clots, the difference in the average pore size value was only 1.3 μm and the difference in the % area was only 2.5% between room temperature and 37℃. Therefore, the difference is likely due to a difference in the number of samples measured, rather than due to differences in the temperature.

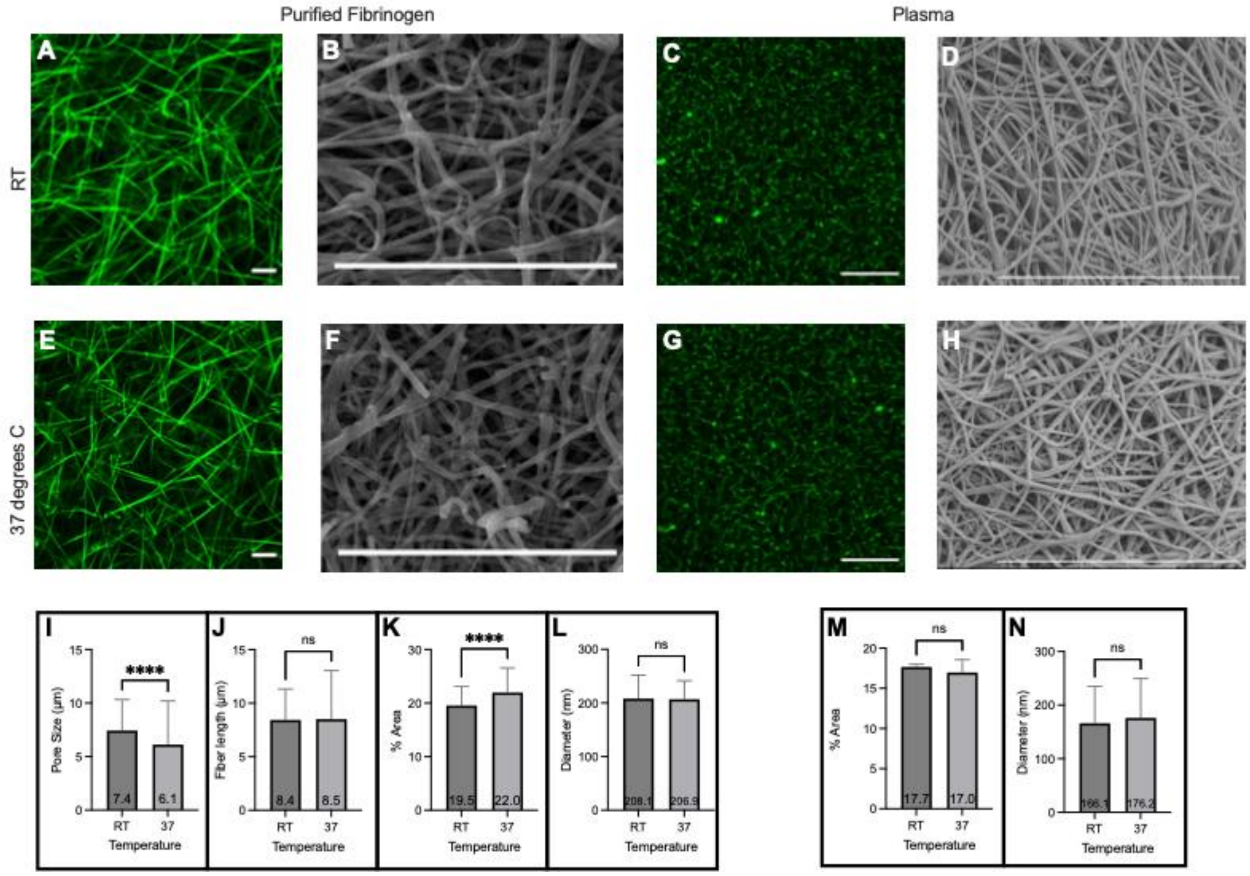

Fig. S.2: Clot structures at room temperature vs 37℃. Confocal microscopy images were taken of clots made with purified fibrinogen (A, E) and plasma (C, G) at room temperature or 37℃, respectively. Pore size (I), fiber length (J), and percent area covered (K) were measured for clots made with purified fibrinogen using confocal microscopy images; percent area covered was measured for plasma clots (M). Scanning electron microscopy images were taken of clots made with purified fibrinogen or plasma at 20,000x at RT (B, D) and 37℃ (F, H); diameter was measured (L, N); scale bars 10 μm.

S.4 Raw Turbidity/Turbidimetry Datasets

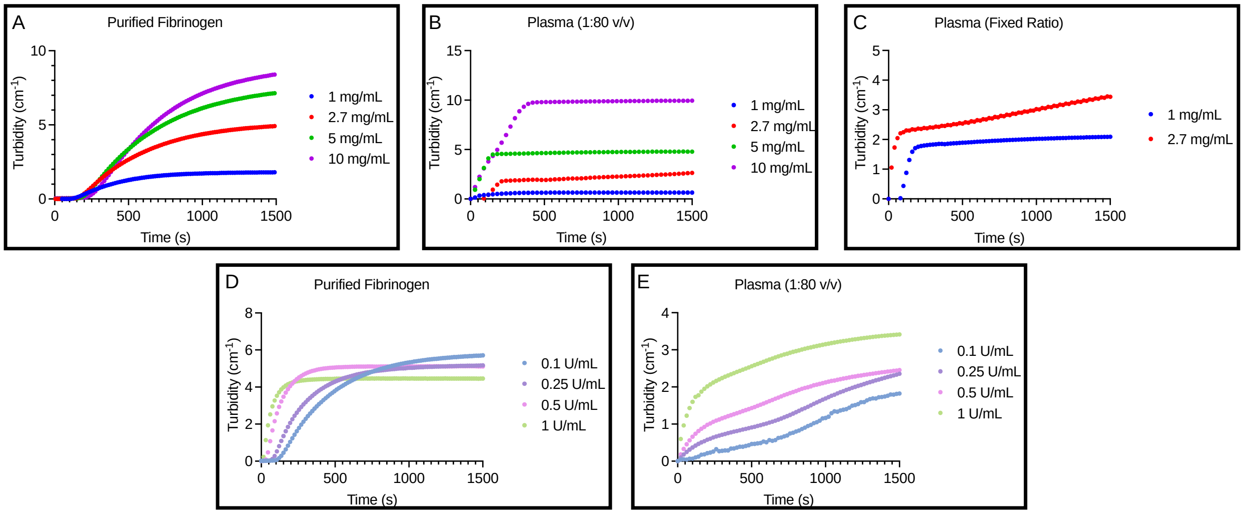

Fig. S.3: Raw Turbidity Datasets. Turbidity polymerization curves over time for A) purified fibrinogen, B) plasma with a 1:80 volume ratio of tissue factor, and C) plasma with a fixed ratio of fibrinogen to tissue factor, with fibrinogen concentrations of 1, 2.7, 5, and 10 mg/mL. Turbidity polymerization curves over time for D) purified fibrinogen and E) plasma, with thrombin concentrations of 0.1, 0.25, 0.5, and 1 U/mL.

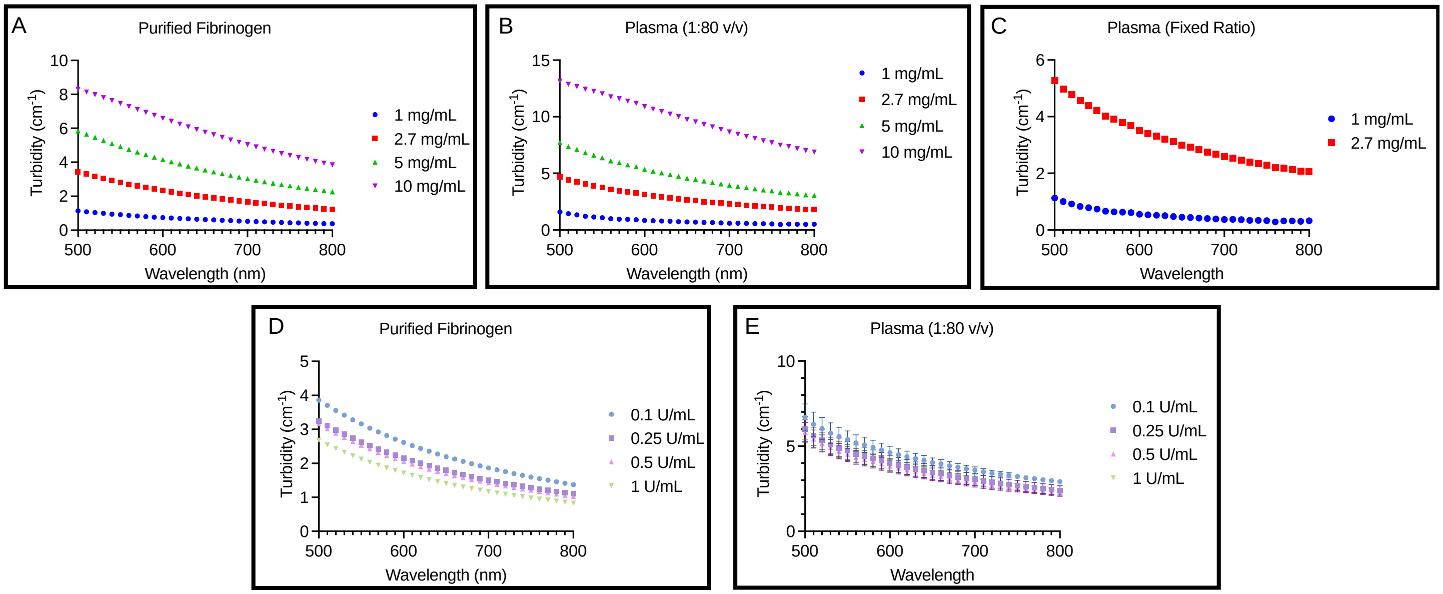

Fig. S.4: Raw Turbidimetry Datasets. Turbidity versus wavelength curves for A) purified fibrinogen with 0.1 U/mL thrombin, B) plasma with a 1:80 volume ratio of tissue factor, and C) plasma with a fixed ratio of fibrinogen to tissue factor, with fibrinogen concentrations of 1, 2.7, 5, and 10 mg/mL. Turbidity versus wavelength curves for D) purified fibrinogen and E) plasma, with thrombin concentrations of 0.1, 0.25, 0.5, and 1 U/mL.

S.5 Fixed Ratio of Tissue Factor to Fibrinogen

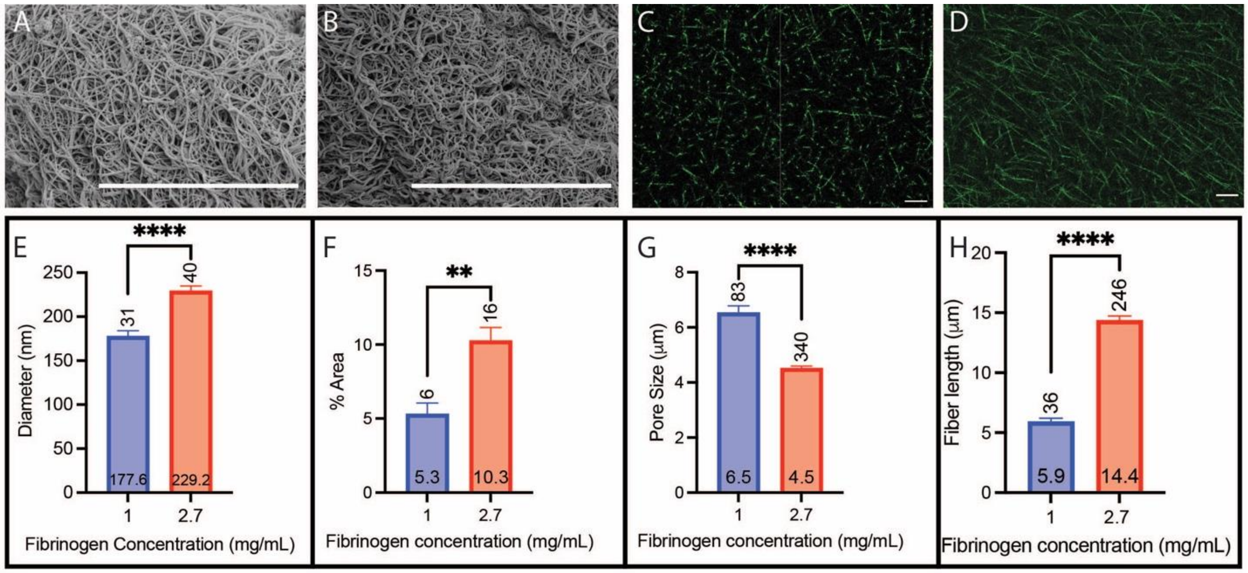

Fig. S.5: Fixed Ratio of Tissue Factor to Fibrinogen. Plasma microscopy with fixed ratio of fibrinogen to tissue factor A) SEM images obtained on a Zeiss SIGMA at 20 kx magnification on clots made with A) 1 mg/mL and B) 2.7 mg/mL fibrinogen. C-D) Confocal images obtained on a Zeiss LSM800 using a 63x oil immersion objective on clots made with C) 1 mg/mL (brightened for visualization with FIJI), and D) 2.7 mg/mL fibrinogen. E) Diameters acquired from SEM images, F) Pore sizes acquired from confocal images, G) Percent fluorescent density obtained from confocal images, H) Fiber lengths obtained from confocal images. (Scale bars 10 µm; ** $p<0.01$, **** $p<0.0001$).

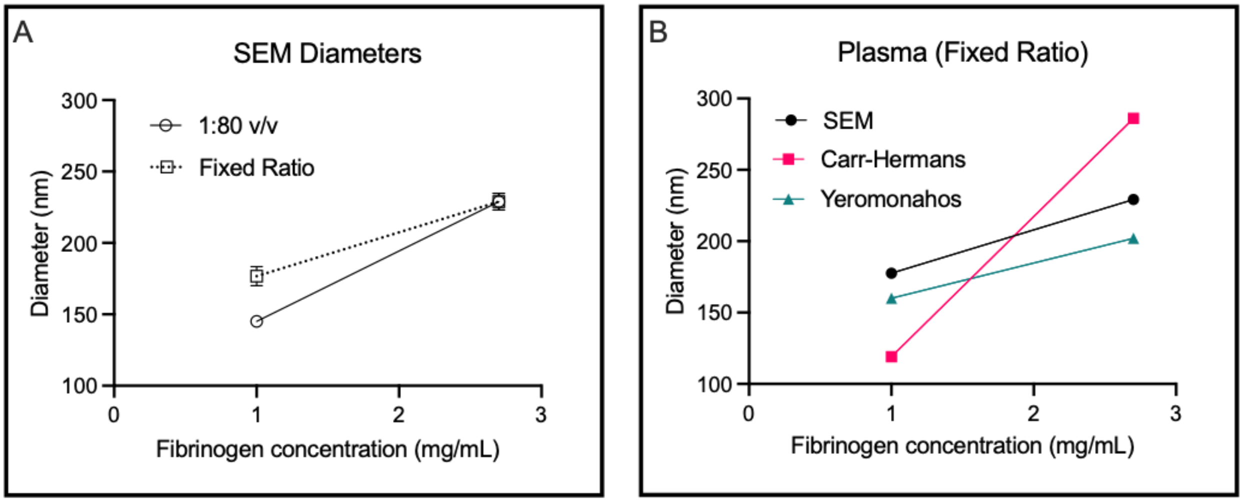

Fig. S.6: Diameters for fixed ratio of TF to fibrinogen. Diameter values obtained A) from SEM imaging for plasma containing a fixed ratio of fibrinogen to tissue factor and plasma containing a 1:80 volume ratio of tissue factor and B) from the Carr-Hermans and Yeromonahos turbidimetric approaches, as well as from SEM imaging for plasma containing a fixed ratio of fibrinogen to tissue factor for fibrinogen concentrations of 1 and 2.7 mg/mL.

For the plasma samples made with a fixed ratio of TF:fibrinogen, there is a statistically insignificant difference in the time it takes to start clot formation with similar trends for both conditions (Table S.1). As expected, the 1 mg/mL sample takes longer to form, forms at a slower rate, and has a higher maximum turbidity compared to the 2.7 mg/mL sample. For a fixed ratio of TF to fibrinogen, the lag time is slightly longer, the rate of formation is faster, and maximum turbidity is larger when compared to when there is a v/v ratio for 1 mg/mL fibrinogen.

	Fixed Ratio Plasma		
Fibrinogen Concentration (mg/mL)	1	2.7	p-value
Lag Time (s)	80 ± 0	45 ± 22	ns
Rate of Clot Formation (cm^{-1}/s $x10^{-3}$)	21 ± 1	39 ± 3	**
Maximum Turbidity (cm^{-1})	2.15 ± 0.15	4.16 ± 0.19	****

Table S.1: The lag phase, rate of clot formation, and max turbidity values for plasma containing a fixed ratio of fibrinogen to tissue factor with fibrinogen concentrations of 1 mg/mL and 2.7 mg/mL; ns not significant, ** p<0.01, **** p<0.0001. A standard deviation of 0 corresponds to all of the values being the same.

S.6 Trends with Maximum Turbidity

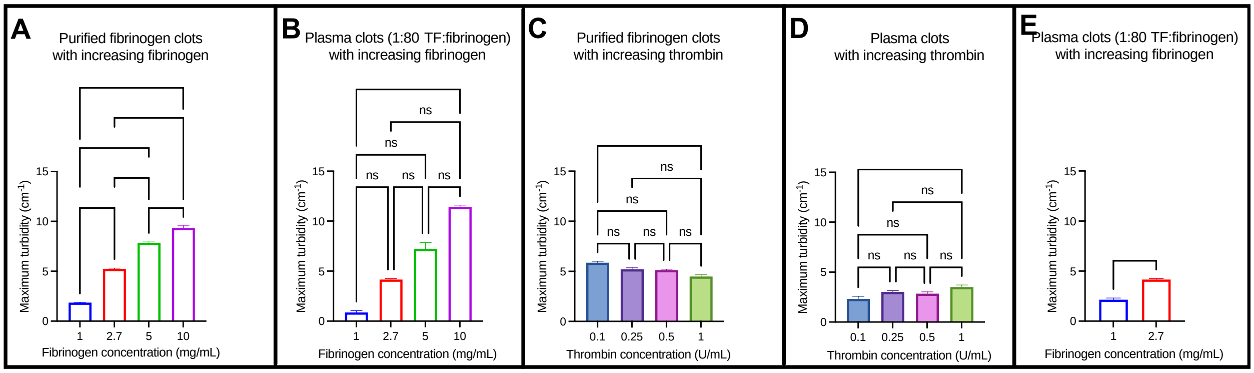

Fig. S.7. Increasing fibrinogen and thrombin and maximum turbidity.

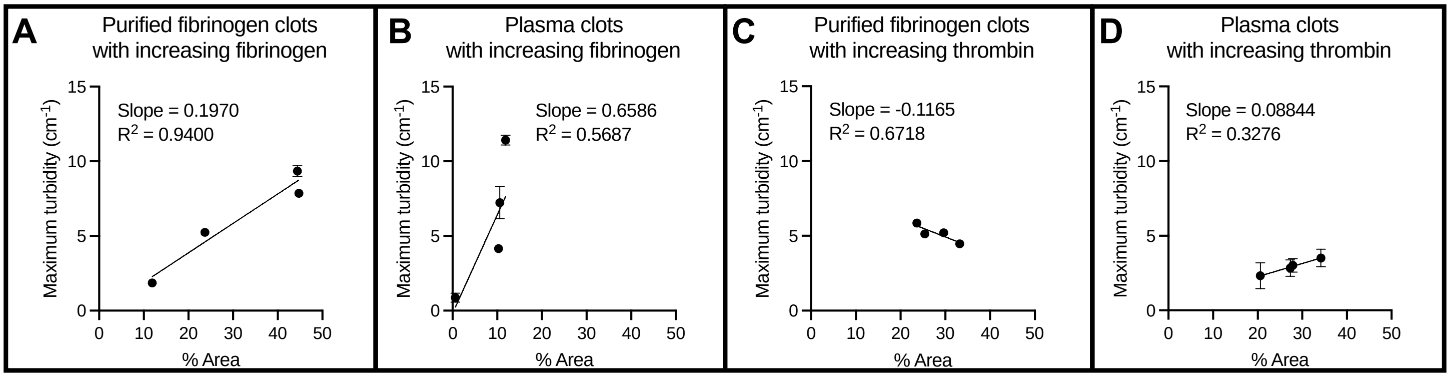

Fig. S.8. Increasing % area and maximum turbidity.

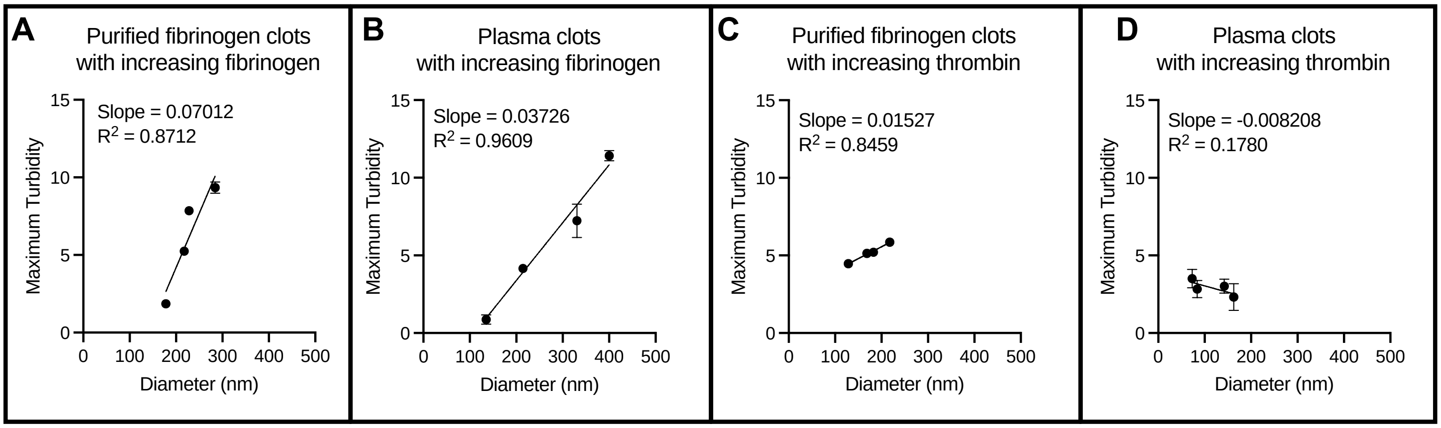

Fig. S.9. Increasing diameter and maximum turbidity.

S.7 General Trends

	Purified Fibrinogen		Plasma	
	Increasing Fibrinogen	Increasing Thrombin	Increasing Fibrinogen	Increasing Thrombin
SEM Diameter	↑	↓	↑	↓
Pore Size	↓	↓	↓	↓
% Area	↑	↑	↑	↑
Fiber Length	↓	↓	↓	↓
Lag Time	↑	↓	↓	↓
Rate of Formation	↑	↑	↑	↓
Maximum Turbidity	↑	↓	↑	↑
Carr-Hermans Diameter Approximation	↑	↓	↑	↓
Yeromonahos Diameter Approximation	↑	↓	↑	↓

Table S.2: Overall trends found throughout this work. Red arrows denote differences between purified fibrinogen and plasma. Upward facing arrows represent increasing parameter values with increasing fibrinogen/thrombin concentrations, downward facing arrows represent decreasing parameter values with increasing fibrinogen/thrombin concentrations.

	Purified Fibrinogen				Plasma (Thrombin)		
Parameter	Intercept	*[Fibrinogen]	*[Thrombin]	R^2	*[Fibrinogen]	*[Thrombin]	R^2
Diameter	185.46	10.89	-88.73	0.9691	59.89	-100.15	0.8163
Pore Size	8.27	-0.68	-4.45	0.8113	3.03	-3.59	0.5990
% Area	14.27	3.46	8.99	0.7133	7.98	12.87	0.8346
Fiber Length	9.53	-0.66	-3.95	0.8543	5.29	-5.62	0.6652
Lag Time	118.81	10.77	-168.08	0.8681	30.61	-97.62	0.5639
Rate of Formation	3.61	0.42	30.78	0.9344	0.85	-0.89	0.8194
Max Turbidity	3.10	0.71	-0.37	0.8023	0.89	1.10	0.7654

Table S.3. Coefficients for multiple linear regressions for clots made with purified fibrinogen (purple) and plasma (blue) with varying fibrinogen and thrombin concentrations. The format of the equation is: Parameter = X*[Fibrinogen] + Y*[Thrombin] + Intercept. Red text indicates values that have opposite sign (positive or negative) to its counterpart.

	Plasma (TF)				Plasma (Thrombin)		
Parameter	Intercept	*[Fibrinogen]	*[TF]	R^2	*[Fibrinogen]	*[Thrombin]	R^2
Diameter	154.26	28.21	-0.18	0.9146	59.89	-100.15	0.8163
Pore Size	7.47	-0.21	-0.03	0.7926	3.03	-3.59	0.5990
% Area	4.08	0.84	0.01	0.4721	7.98	12.87	0.8346
Fiber Length	1.10	-0.64	0.20	0.9329	5.29	-5.62	0.6652
Lag Time	95.37	0.03	-0.56	0.5550	30.61	-97.62	0.5639
Rate of Formation	18.29	-1.41	0.15	0.1246	0.85	-0.89	0.8194
Max Turbidity	1.17	1.11	-0.00	0.9677	0.89	1.10	0.7654

Table S.4. Coefficients for multiple linear regressions for clots made with plasma with either TF (green) or thrombin (blue, same as Table S.3). The format of the equation is: Parameter = X*[Fibrinogen] + Y*[TF] + Intercept; Parameter = X*[Fibrinogen] + Y*[Thrombin] + Intercept. Red text indicates values that have opposite sign (positive or negative) to its counterpart.
